# Case Report: Novel Compound Heterozygous *RNASEH2B* Mutations Cause Aicardi–Goutières Syndrome

**DOI:** 10.3389/fimmu.2021.672952

**Published:** 2021-04-26

**Authors:** Jessica Garau, Silvia Masnada, Francesca Dragoni, Daisy Sproviero, Federico Fogolari, Stella Gagliardi, Giana Izzo, Costanza Varesio, Simona Orcesi, Pierangelo Veggiotti, Gian Vincenzo Zuccotti, Orietta Pansarasa, Davide Tonduti, Cristina Cereda

**Affiliations:** ^1^ Genomic and Post-Genomic Unit, IRCCS Mondino Foundation, Pavia, Italy; ^2^ Unit of Pediatric Neurology, V. Buzzi Children’s Hospital, Milan, Italy; ^3^ C.O.A.L.A (Center for Diagnosis and Treatment of Leukodystrophies), V. Buzzi Children’s Hospital, Milan, Italy; ^4^ Department of Biology and Biotechnology “L. Spallanzani”, University of Pavia, Pavia, Italy; ^5^ Department of Mathematics, Computer Science and Physics, University of Udine, Udine, Italy; ^6^ Department of Pediatric Radiology and Neuroradiology, V. Buzzi Children’s Hospital, Milan, Italy; ^7^ Department of Child Neurology and Psychiatry, IRCCS Mondino Foundation, Pavia, Italy; ^8^ Department of Brain and Behavioral Sciences, University of Pavia, Pavia, Italy; ^9^ Department of Biomedical and Clinical Science “L. Sacco, University of Milan, Milan, Italy; ^10^ Department of Pediatrics, V. Buzzi Children’s Hospital, Milan, Italy

**Keywords:** Aicardi–Goutières Syndrome, next generation sequencing, *RNASEH2B*, genetics, novel mutation

## Abstract

Aicardi–Goutières Syndrome (AGS) is a rare disorder characterized by neurological and immunological signs. In this study we have described a child with a phenotype consistent with AGS carrying a novel compound heterozygous mutation in *RNASEH2B* gene. Next Generation Sequencing revealed two heterozygous variants in *RNASEH2B* gene. We also highlighted a reduction of RNase H2B transcript and protein levels in all the family members. Lower protein levels of RNase H2A have been observed in all the members of the family as well, whereas a deep depletion of RNase H2C has only been identified in the affected child. The structural analysis showed that both mutations remove many intramolecular contacts, possibly introducing conformational rearrangements with a decrease of the stability of RNase H2B and strongly destabilizing the RNase H2 complex. Taken together, these results highlight the importance of an integrated diagnostic approach which takes into consideration clinical, genetic, and molecular analyses.

## Introduction

Aicardi–Goutières Syndrome (AGS) is a rare pediatric disorder characterized by both neurological and immunological signs such as leukodystrophy, cerebral atrophy, and calcifications, lymphocytosis and increased levels of interferon-alpha (1). Mutations in nine genes have been described as causative of AGS (2, 3), and they all lead to the pathological activation of the innate immune system usually observed in these patients (3, 4). In the worldwide cohort of patients, as well as in the Italian one, the most frequently mutated gene is *RNASEH2B * (2, 5). *RNASEH2B* encodes one of the three subunits of RNase H2, one of the major endonucleases in humans (6). Loss of function mutations of this gene lead to the accumulation of undigested endogenous nucleic acids that are able to trigger the abnormal innate immune response typical of this disorder (7). Here we describe for the first time the case of a child affected by AGS with a novel compound heterozygous mutation in the splice acceptor site within intron 1 and in exon 4 of *RNASEH2B* gene.

## Case Presentation

He is a boy born from healthy unrelated parents, after uneventful pregnancy and delivery. He presented at 12 months of age with irritability, sterile pyrexia, neuromotor regression with loss of trunk and head control. Neurological examination revealed marked irritability, startle reaction to even mild sensory stimuli, acquired microcephaly, and pyramidal and extrapyramidal signs. Other features of AGS (*i.e.* chilblains, seizures, hepatic dysfunction) were not present. MRI showed white matter hyperintensity involving periventricular and deep regions and slight cerebral atrophy (**Figure S1**). Lumbar puncture showed pleocytosis [16 lymphocytes/mm (3)]. Interferon alpha levels were normal in the Cerebrospinal fluid (CSF); neopterin, biopterin, and folates were not measured. The possibility of acquired acute encephalitis was initially considered. Nevertheless, the presence of cerebral atrophy and microcephaly at onset leads to expanding the differential diagnosis. AGS was suspected, therefore, the boy underwent a head CT scan, but no calcifications were observed. Serologies for TORCH complex and HIV were all negative. Interferon alpha level was not evaluated but blood interferon signature was positive (6.862; normal range: 0–2.22). AGS diagnostic criteria (8) were partially fulfilled (**Table S1**), and finally molecular analysis confirmed the clinical suspicion. Once the diagnosis was done, JAK inhibitors were initiated. Patient follow-up and response to treatment were described in Mura et al. (9)

**Figure 1:**
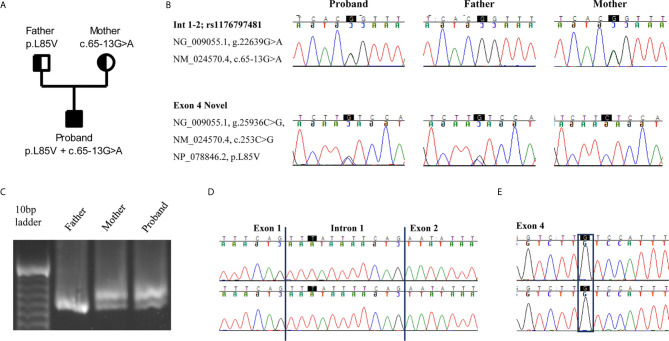
An intron variant and a novel missense mutation in *RNASEH2B* gene are identified. **(A)** Pedigree of the family. Squares and circles represent male and female family members, respectively. **(B)** The two variants c.65-13G>A and c.253C>G are confirmed in the proband and parents by Sanger sequencing. **(C)** cDNAs from the proband and his parents were resolved on a 3% agarose-gel, showing the different splice variants. **(D, E)** Electropherograms of the two splice variants identified in the proband. The highest peak presents an intron retention due to the c.65-13G>A mutation whereas the lowest one the c.253C>G variant.

Next Generation Sequencing (NGS) revealed two heterozygous variants in *RNASEH2B* gene (NM_024570.4). The first one, c.65-13G>A, was a maternally inherited intronic variant, and it has already been described in the literature (2). The second one was a novel paternally inherited variant that caused the replacement of the Leucine with a Valine, p.L85V. *In silico* prediction software, such as SIFT (http://sift.jcvi.org/), Polyphen2 (http://genetics.bwh.harvard.edu/pph2/), and Mutation Taster (http://www.mutationtaster.org/) define this variant as damaging or disease causing. Gene segregation was studied by trio Sanger sequencing, and both parents were confirmed to be heterozygous carriers of each mutation (**Figures 1A, B** **)**.

Since the intronic mutation is placed in a critical splicing site, we decided to study its effect on *RNASEH2B* transcript. We amplified a cDNA region spanning from exon 1 to exon 5 to investigate different splicing variants. The cDNA derived from patient’s and mother’s RNA, both presenting the c.65-13G>A variant, showed two different transcripts, whereas the father presented only one transcript (**Figure 1C**). To better investigate differences between these two transcripts, we studied their sequences by Sanger sequencing. The upper band in the agarose gel represents a longer transcript that resulted from the retention of 11 nucleotides belonging to intron 1 (**Figure 1D**). The lower band, as well as the only band identified in the father, represents the wild-type transcript, and both father and proband presented the c.253C>G variant in exon 4 (**Figure 1E**).

**Figure 2:**
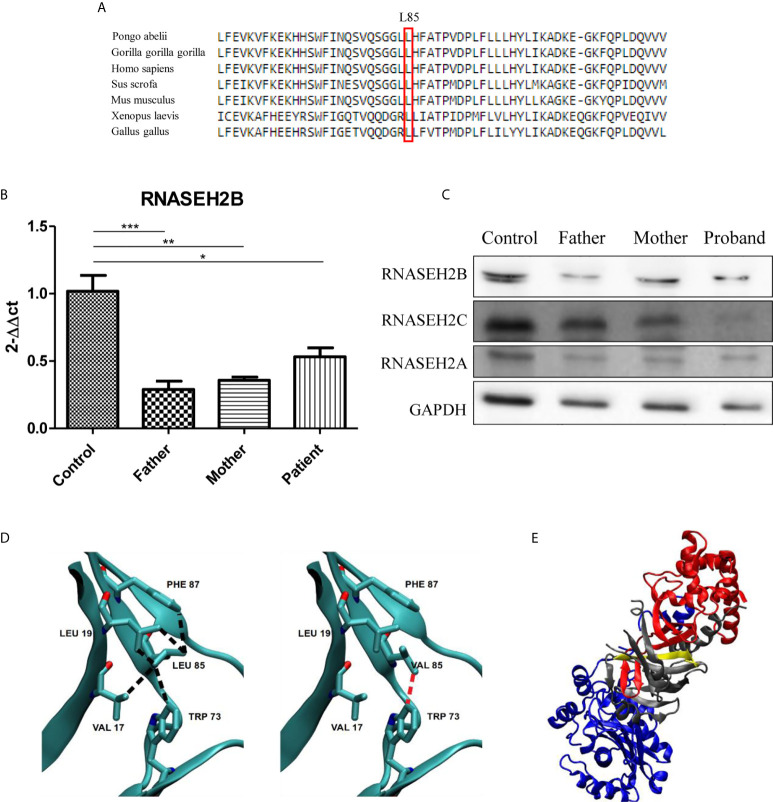
Characterization of the two *RNASEH2B* mutations. **(A)** The L85 residue of *RNASEH2B* is evolutionarily conserved crossing all examined species. The conserved Leucine residue is shown in the rectangular box. Protein sequences were obtained from the National Center for Biotechnology (NCBI). **(B)** Expression levels of *RNASEH2B* transcripts in a healthy control, parents, and proband *P < 0.05, **P < 0.01, ***P < 0.001. **(C)** Western blot analysis of RNase H2B, RNase H2C, and RNase H2A protein levels in a healthy control, parents, and proband. GAPDH has been used as loading control. **(D)** The contacts involving amino acid L85 with V17, L19, W73, and F87 on the native (left panel, black broken lines) and L85V mutant (right panel, red broken lines) of *RNASEH2B*. **(E)** The crystal structure of RNASE H2 complex with RNASEH2A, *RNASEH2B*, RNASEH2C highlighted in blue, red and gray, respectively. On the same figure the first 30 residues of *RNASEH2B* (of which 1–12 are missing in the crystallographic structure) are highlighted in yellow.

Since p.L85V is novel mutation, we decided to perform a comparative amino acid alignment of RNase H2B protein across vertebrates using multiple sequence alignment analysis. This analysis revealed that amino acid L85 is highly conserved during evolution (**Figure 2A**).

We continued our analysis by investigating the effects of both mutations on gene expression and RNase H2 protein levels. We observed a significant decrease of *RNASEH2B* expression in all the members of the family by RT-qPCR (**Figure 2B**). This result was also confirmed by Western blot, which highlighted a reduction of RNase H2B protein levels in the family with a deeper decrease in the father (**Figure 2C**), as already observed for the transcript. Since RNase H2 is a heterotrimeric enzyme, we also investigated the protein levels of the other two subunits. Lower RNase H2A protein levels have been observed in all the members of the family, whereas a strong depletion of RNase H2C was identified only in the affected child (**Figure 2C**).

The analysis of native and homology modeled mutant structures showed that replacement of Leucine by the shorter Valine sidechain eliminates many of the intramolecular contacts with hydrophobic moieties of V17, L19, W73, and F87, besides the intermolecular contact with V144 in RNase H2C (**Figure 2D**). The contact with W73 appeared particularly important because this residue is conserved in all (non-divergent) vertebrates’ sequences. It is likely that the mutation may introduce conformational rearrangements with a decrease of the stability of RNase H2B and possibly of its complex with RNase H2C. The finding that the mutant W73L lowers the stability of RNase H2B (10) supports this conclusion.

At last, the splicing mutation in intron 1 results in the deletion of the first 30 residues of RNase H2B which appears to strongly destabilize the complex because this region occurs within a *β*-strand interfacing RNase H2B and RNase H2C (**Figure 2E**).

## Discussion

Here we report the case of a child diagnosed with AGS caused by one previously described and one novel variant in *RNASEH2B* gene in a compound heterozygous state. *RNASEH2B* has been defined an AGS-causing gene in 2006 (11), and it is usually the most frequently mutated gene found in these patients (2, 5). Patients mutated in *RNASEH2B* are more likely to retain some motor and communication abilities, and they usually develop later-onset forms of AGS with a longer life expectancy (6).

RNase H2 is one of the major sources of ribonuclease activity in eukaryotes (6), and it is composed of three subunits: the catalytic subunit RNase H2A and the two auxiliary subunits RNase H2B and RNase H2C necessary to stabilize and form an enzymatically active heterotrimer (12). All the subunits are all equally important for the enzymatic activity (13).

Mutations in RNase H2 enzyme determine an accumulation of nucleic acids derived from endogenous retroelements or from inefficient removal of ribonucleotides from genomic DNA and an increased DNA damage. All these scenarios lead to an abnormal stimulation of immunological pathways determining an increased IFN-α release, which is one of the main features of AGS (7).

In our patient, the splice site variant in intron 1 has already been reported in literature as a mutation causative of AGS when in a compound heterozygous state (2). Indeed, the mother is a heterozygous carrier of this intronic mutation in *RNASEH2B* gene, and she showed no AGS symptoms. Although c.253C>G/p.(Leu85Val) variant has never been described, we inferred its pathogenicity from the results of *in vitro* and *in silico* analyses. Both mutations lead to a decreased expression of RNase H2B transcript and to reduced protein levels of all RNase H2 subunits suggesting an alteration of the enzyme main functions. It has been described in literature that mutations or deletions of one of the three subunits have an effect also on the other two unmutated subunits (14, 15). This overall RNase H2 subunits protein levels decrease was clearer in the affected patient, who present both mutations and, therefore, a pathological phenotype. The two parents, who are heterozygous carriers and, therefore, present no AGS-related symptoms, showed a lower reduction of RNase H2A and RNase H2C protein levels that does not affect enzyme functionality. Taken together, these results suggest that both mutations are needed to determine a pathological phenotype. All these results were also confirmed by *in silico* analyses on the available crystallographic native and homology modeled mutant structures. The splice variant in intron 1 results in the loss of an amino acidic region at the interface between RNase H2B and RNase H2C, strongly destabilizing the complex. L85V mutation reduces the number of favourable intramolecular hydrophobic interactions leading to a likely destabilization of RNase H2B and possibly, through related interactions, of its complex with RNase H2C.

In conclusion, our study presented novel compound heterozygous mutations in *RNASEH2B* gene in a child with a phenotype consistent with AGS. These results highlight the importance of an integrated diagnostic approach which take into consideration both clinical and genetic analyses.

## Ethics Statement

The studies involving human participants were reviewed and approved by Local ethics committee (approval n. 3549/2009 of 30/9/2009 and 11/12/2009, and n.20170035275 of 23/10/2017) of the IRCCS Mondino Foundation, Pavia. Written informed consent to participate in this study was provided by the participants’ legal guardian/next of kin. Written informed consent was obtained from the minor(s)’ legal guardian/next of kin for the publication of any potentially identifiable images or data included in this article.

## Author Contributions

Conceptualization: JG, SM, DS, DT, and CC. Formal analysis: JG, FD, DS, FF, OP, and CC. Funding acquisition: SO and CC. Investigation: JG, SM, FD, DS, FF, GI, and DT. Methodology: JG, DS, FF, SG, OP, DT, and CC. Resources: DT, OP, and CC. Supervision: DT and CC. Visualization: JG, SM, DS, SG, OP, and DT. Writing—original draft: JG, SM, DS, SG, OP, CV, SO, DT, and CC. Writing—review and editing: SO, PV, GZ, OP, DT, and CC. All authors contributed to the article and approved the submitted version.

## Funding

This study was supported by NIH-funded grants (U01NS106845-01A1) (The content is solely the responsibility of the authors and does not necessarily represent the official views of the National Institutes of Health) and by the Italian Health Ministry grant RC 2017-2020 to IRCCS Mondino Foundation, Pavia, Italy.

## Conflict of Interest

The authors declare that the research was conducted in the absence of any commercial or financial relationships that could be construed as a potential conflict of interest.
