# Supplementary material for: Case Report: Novel Compound Heterozygous RNASEH2B Mutations Cause Aicardi–Goutières Syndrome
Source: Front Immunol. 2021 Apr 26;12:672952. doi: 10.3389/fimmu.2021.672952 (PMC8107470; doi:10.3389/fimmu.2021.672952)
Supplement: Supplementary file 1 [file DataSheet_1.docx]

Supplementary Material

**MATERIALS AND METHODS**

**DNA isolation and Next Generation Sequencing**

Peripheral blood samples were collected in EDTA tubes after signed an informed consent form for genetic analysis. Genomic DNA has been isolated using the Maxwell^®^ 16 System DNA Purification (Promega, USA). DNA was quantified with NanoDrop ND1000 UV-Vis Spectrophotometer and Qubit^®^ fluorometer (Thermo-Fisher Scientific, USA). The genetic test of the proband has been performed using Nextera Enrichment Sample Illumina (Illumina, USA) and the gene panel including AGS-related genes as previously described^1^. All identified variants and gene segregation were confirmed by Sanger sequencing.

**RNA isolation and cDNA sequencing**

Peripheral blood samples were collected into PaxGene™ tubes (PreAnalytiX, Switzerland) and RNA isolation was performed according to the manufacturer’s protocol. RNA concentrations were assessed with NanoDrop ND1000 UV-Vis Spectrophotometer (Thermo-Fisher Scientific, USA). 1µg of RNA was retrotranscribed using the iScript™ Reverse Transcription Supermix (Bio-Rad, USA). The cDNA has been amplified by PCR and then all the sample were loaded on a 3% agarose gel with ethidium bromide. cDNA bands were cut out of the agarose gel and cDNA samples were purified using NucleoSpin Gel and PCR Clean‑up (Macherey-Nagel, Germany). cDNA was then sequenced by Sanger sequencing.

**Real-time quantitative PCR (RT-qPCR)**

For *RNASEH2B* analysis, qPCR reactions included 200 nM of each oligonucleotide, 1 μL of SYBR Green SuperMix (BioRad, USA) and 1 μL of cDNA template. The following primers were used: RNASEH2B F: GCCAAATCCTCCATCAAAGA; RNASEH2B R: TGAGCTGCAGTCATTTTGCT. Mean Cycle threshold (Ct) values were normalized against those determined for *GAPDH*. Fold-expression differences relative to the healthy control were determined using the 2^-ΔΔCt^ method^2^. Significance of gene expression changes relative to the control was analysed using one-way ANOVA and the Tukey’s post hoc analysis using Prism GraphPad 5.0 software. P < 0.05 was considered statistically significant.

**Interferon Signature**

The expression analysis of six interferon-stimulated genes was performed using the TaqMan Universal PCR Master Mix (Applied Biosystems, UK), and cDNA derived from 40 ng total RNA previously isolated from peripheral blood. The relative abundance of target transcripts was measured using TaqMan probes for *IFI27*, *IFI44L*, *IFIT1*, *ISG15*, *RSAD2* and *SIGLEC1*, and normalized to the expression level of *HPRT1* and *18S* as described in literature^3^. The interferon score was calculated as described in Garau et al., 2019^1^.

**Peripheral blood mononuclear cells (PBMCs) and protein isolation**

PBMCs were isolated from peripheral venous blood conserved in EDTA tubes using Histopaque®-1077 (Sigma-Aldrich, USA) and following manufacturer’s specifications. Soluble protein samples were obtained from PBMCs with the extraction using RIPA buffer. Protein concentration was determined using bicinchoninic acid method (Sigma-Aldrich, USA) and bovine serum albumin (Sigma-Aldrich, USA) as standard.

**Western Blot**

Samples containing 25 μg of proteins were loaded into 12.5% SDS-PAGE gel and transferred to nitrocellulose membranes using a semidry transfer apparatus (Trans-blot Turbo, Bio-Rad, USA). Membranes were blocked with 5% of non-fat dry milk in 1X TBS-T buffer for 1h and incubated overnight with the primary antibodies at 4°C. Immunoreactivity was detected using the donkey anti-rabbit or anti-mouse secondary peroxidase-conjugated (GE Healthcare, UK) and the enhanced chemiluminescence detection kit (ECL Advance, GE Healthcare, UK). The following antibodies were used: rabbit polyclonal anti-RNase H2A (ab83943, Abcam, UK, dilution 1:1000), rabbit polyclonal anti-RNase H2C (16518-1-AP, Proteintech, USA, dilution 1:1000), rabbit polyclonal anti-RNase H2B (kindly provided by Prof. Muzi-Falconi^4^, dilution 1:500) and rabbit polyclonal anti-GAPDH (GTX100118, GeneTex, USA, dilution 1:10000).

**Protein structure modelling**

The structure of human RNase H2 complex is available from the Protein Data Bank (www.rcsb.org) with PDB id. code 3PUF^5^. To generate the mutant structure residue L85 in chain B was mutated *in silico* to valine. Missing sidechains were added using DeepView 4.10^6^. Any distance between atoms, from two different residues, shorter than the sum of their van der Waals radii plus 1 Å was indicative of a contact between two residues^7^.

**SUPPLEMENTARY FIGURE**

**
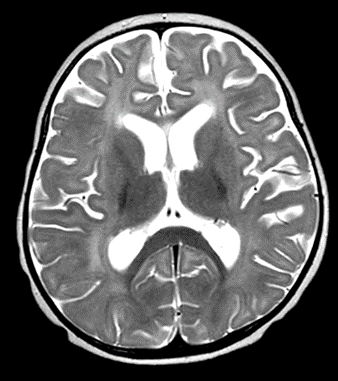
**

**Figure S1.** Axial T2wi at onset showing mild cerebral atrophy white matter hyperintensity mainly involving periventricular and deep white matter regions.

**Table S1.** AGS diagnostic criteria^8^ fulfilled by the patient^9^.

| **Fulfilled AGS diagnostic criteria** |
| --- |
| - Early onset encephalopathy with psychomotor delay, spasticity, extrapyramidal signs and microcephaly  - Cerebral white matter abnormalities  - Cerebral atrophy  - Exclusion of pre−/perinatal infections, in particular the TORCH complex  - Chronic lymphocytosis (> 5 cells/mm3) on CSF examination, not accompanied by any other sign of an infectious process  - Important systemic symptoms in the early stages of the disease include irritability, feeding and sleeping difficulties, unexplained fevers |
| **Not fulfilled AGS diagnostic criteria** |
| - Calcifications particularly visible at basal ganglia level (putamen, pallidus and thalamus), but also extending to the periventricular white matter  - Raised INF-alpha in the CSF (> 2 IU/ml)  - Chilblain-like skin lesions on the fingers, toes and ears |

**References**

1. Garau J, Cavallera V, Valente M, et al. Molecular Genetics and Interferon Signature in the Italian Aicardi Goutières Syndrome Cohort: Report of 12 New Cases and Literature Review. *J Clin Med* (2019) 26;8(5):750. doi: 10.3390/jcm8050750.

2. Pfaffl MW. A new mathematical model for relative quantification in real-time RT-PCR. *Nucleic Acids Res* (2001) 29(9):e45. doi: 10.1093/nar/29.9.e45.

3. Rice GI, Forte GM, Szynkiewicz M, et al. Assessment of interferon-related biomarkers in Aicardi-Goutières syndrome associated with mutations in TREX1, RNASEH2A, RNASEH2B, RNASEH2C, SAMHD1, and ADAR: a case-control study. *Lancet Neurol* (2013) 12(12):1159-69. doi: 10.1016/S1474-4422(13)70258-8.

4. Pizzi S, Sertic S, Orcesi S, et al. Reduction of hRNase H2 activity in Aicardi–Goutières syndrome cells leads to replication stress and genome instability. *Hum Mol Genet* (2015) 24(3):649-58. doi: 10.1093/hmg/ddu485.

5. Figiel M, Chon H, Cerritelli SM, Cybulska M, Crouch RJ, Nowotny M. The Structural and Biochemical Characterization of Human RNase H2 Complex Reveals the Molecular Basis for Substrate Recognition and Aicardi-Goutières Syndrome Defects. *J Biol Chem* (2011) 286(12):10540-50. doi: 10.1074/jbc.M110.181974.

6. Schwede T, Kopp J, Guex N, Peitsch MC. SWISS-MODEL: An automated protein homology-modeling server. *Nucleic Acids Research* (2009) 31(13),3381–3385. doi: 10.1093/nar/gkg520

7. Berrera M, Molinari H, Fogolari F. Amino acid empirical contact energy definitions for fold recognition in the space of contact maps. *BMC Bioinformatics* (2003) 4:8. doi: 10.1186/1471-2105-4-8.

8. Tonduti D, Panteghini C, Pichiecchio A, et al. Encephalopathies with intracranial calcification in children: clinical and genetic characterization. *Orphanet J Rare Dis* (2018) 13(1):135. doi: 10.1186/s13023-018-0854-y.

9. Mura E, Masnada S, Antonello C, et al. Ruxolitinib in Aicardi-Goutières syndrome. *Metab Brain Dis* (2021). doi: 10.1007/s11011-021-00716-5.
